# Long-term effects of *H*. *pylori* eradication on epigenetic alterations related to gastric carcinogenesis

**DOI:** 10.1038/s41598-018-32717-3

**Published:** 2018-09-25

**Authors:** Yuki Michigami, Jiro Watari, Chiyomi Ito, Keisuke Nakai, Takahisa Yamasaki, Takashi Kondo, Tomoaki Kono, Katsuyuki Tozawa, Toshihiko Tomita, Tadayuki Oshima, Hirokazu Fukui, Takeshi Morimoto, Kiron M. Das, Hiroto Miwa

**Affiliations:** 1 Division of Gastroenterology, Department of Internal Medicine, Hyogo College of Medicine, Nishinomiya, Japan; 2 Department of Clinical Epidemiology, Hyogo College of Medicine, Nishinomiya, Japan; 3 Division of Gastroenterology and Hepatology, Departments of Medicine and Pathology, Robert Wood Johnson Medical School, Rutgers, Cancer Institute of New Jersey, New Brunswick, United States

## Abstract

The risk of gastric cancer (GC) remains in precancerous conditions, including atrophic mucosa and intestinal mucosa (IM), even after *H*. *pylori* treatment. To define the molecular changes following *H*. *pylori* eradication, molecular alterations in the gastric mucosa with and without GC were evaluated in a long-term follow-up study. A total of 232 biopsy specimens from 78 consecutive patients, including atrophic gastritis patients with follow-up ≥3 y after successful *H*. *pylori* eradication (AG group), patients who developed early GC after successful eradication (≥3 y) (GC group), and patients with *H*. *pylori*-positive atrophic gastritis (*Hp* group), were analyzed. *H*. *pylori* eradication was associated with significant reductions of methylation of several genes/loci in atrophic mucosa (non-IM), but not in IM. In contrast, the incidence of CpG island methylator phenotype (CIMP) in IM was significantly higher in the GC group than in the AG group. *miR-124a-3* methylation and *miR-34c* methylation were more frequently identified in IM, with very few in non-IM mucosa among the three groups. *H*. *pylori* eradication can reverse methylation only in non-IM mucosa. CIMP in IM may have potential as a surrogate maker of GC development, and methylation of *miR-124a-3* and *miR-34c* is a molecular event in IM that may not be associated with GC development.

## Introduction

It has been postulated that *Helicobacter pylori* (*H*. *pylori*) infection causes chronic gastritis, comprised of atrophic gastritis, usually with intestinal metaplasia (IM), dysplasia, and gastric cancer (GC). The stepwise nature of this infection, which usually continues over decades, has been defined as a sequence of histological events that confers an increasing risk of malignant transformation, as described in Correa’s hypothesis^1^. In Japan, therefore, national insurance has covered eradication therapy in patients with endoscopically diagnosed chronic gastritis caused by *H*. *pylori* infection since 2013 to prevent the development of GC^2^. Similarly, the International Agency for Research on Cancer Working Group Report in 2014 recommended that all countries explore the possibility of introducing population-based *H*. *pylori* screening and treatment programs as a strategy for GC prevention^3^.

Meta-analyses showed that *H*. *pylori* eradication seems to reduce GC risk^4,5^, and the magnitude of the protective effect is greater among individuals with higher baseline GC risk, such as patients who have undergone endoscopic resection (ER) of early GC^5^. However, long-term studies from Japan showed that even after *H*. *pylori* eradication, the risk of the development of GC remains, and the risk persists in the background of gastric atrophic mucosa, including IM^6,7^. One of the reasons for this may be that the improvement of these precancerous conditions requires several to ten years following *H*. *pylori* treatment^8–10^.

GC develops through the accumulation of genetic and epigenetic alterations. To date, many studies have reported that several molecular alterations, including genetic instability and promoter hypermethylation of multiple tumor-related genes, are associated with GC and precancerous conditions of the stomach^11–25^. In addition, several cancer risk biomarkers were discussed in these studies. Currently, it is considered that dysregulation of noncoding RNAs, e.g. microRNAs (miRNAs) such as methylation of *miR-124a-3* and *miR-34b/c*, also plays important roles in the pathogenesis of GC^24,26–29^. Although some studies evaluated the changes in molecular alterations after *H*. *pylori* eradication, the follow-up period seemed short, mostly within 1 year^14–16,18,19,22,25^ and for less than a mean of 3 years^23,24^. Japanese investigators have recently reported that *miR-124a-3* methylation is an informative marker for predicting the risk of metachronous GC in patients followed for 3 to 5 years after ER of early GC^24,26^. In 2008, a randomized, controlled trial from Japan showed that *H*. *pylori* eradication after ER of early GC could prevent the development of metachronous GC for up to 3 years^30^. However, subsequent long-term follow-up studies from Japan demonstrated that *H*. *pylori* eradication did not reduce the incidence of metachronous GC for more than 5 years after ER of GC^31,32^. Taken together, a long-term study of at least more than 3 years may be required to clarify the chemopreventive effects of *H*. *pylori* eradication on molecular alterations.

In this study, the long-term effects of *H*. *pylori* eradication on genetic and epigenetic molecular alterations including dysregulation of miRNAs and monoclonal antibody (mAb) Das-1, which is strongly associated with GC^22,25,33,34^, were investigated in atrophic mucosa (non-IM) and IM (precancerous conditions) in patients with and without early GC (primary endpoints). The molecular markers linked to carcinogenesis risk in such patients (secondary endpoints) were also determined.

## Results

### Patients’ characteristics

The characteristics of the patients are shown in Table 1. The median duration post-eradication was 5 years (3–7 years) in the atrophic gastritis (AG) group and 5 years (4–9 years) in the GC group; there was no significant difference between the two groups. The mean duration was 6.0 ± 3.1 years in the AG group and 7.0 ± 4.5 years in the GC group. There were also no significant differences in mean age and sex among the three groups.

**Table 1:**
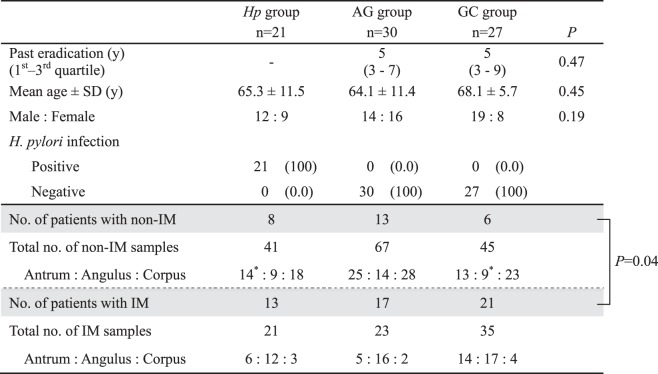
Patients’ characteristics. AG, atrophic gastritis; GC, gastric cancer; IM, intestinal metaplasia. *One sample in the *Hp* group and one sample in the GC group could not be analyzed due to a small sample.

In addition, no significant difference in the number of patients with and without IM among the three groups was seen, whereas the frequency of IM samples was significantly higher in the GC group (43.8%, 35 of 80) than in the *H*. *pylori* (*Hp*, control) (33.9%, 21 of 62) and AG (25.5%, 23 of 90) groups (*P* = 0.04).

### MSI and epigenetic alterations

#### Differences of molecular alterations among the three parts of the stomach in each group

In patients with non-IM, there were no significant differences of molecular events among the three sites of the stomach in both the *Hp* and AG groups. In the GC group, however, methylated-in-tumor-1 (MINT1), MINT31, and *miR-124a-3* methylation tended to be frequently observed in the antrum to the angulus (*P* = 0.08, *P* = 0.05, and *P* = 0.02, respectively). Also, the CpG island methylator phenotype (CIMP) rate tended to be higher in the angulus (*P* = 0.08) (Table 2).

**Table 2:** Molecular alterations in the three parts of the stomach in patients with non-IM and IM among the *Hp*, AG, and GC groups.

No. of patients with non-IM	*Hp* group (n = 21)							AG group (n = 30)							GC group (n = 27)						
	Antrum		Angulus		Corpus		*P*	Antrum		Angulus		Corpus		*P*	Antrum		Angulus		Corpus		*P*
	14*	(%)	9	(%)	18	(%)		25	(%)	14	(%)	28	(%)		13	(%)	9*	(%)	23	(%)	
MSI	0	(0)	1	(11.1)	0	(0)	0.16	0	(0)	0	(0)	1	(3.6)	0.49	2	(15.4)	0	(0)	2	(8.7)	0.46
CIMP	7	(50.0)	6	(66.7)	13	(72.2)	0.42	0	(0)	0	(0)	0	(0)	—	1	(7.7)	2	(22.2)	0	(0)	0.08
*CDH1*	12	(85.7)	7	(77.8)	18	(100)	0.15	9	(36.0)	6	(42.9)	8	(28.6)	0.64	4	(30.8)	6	(66.7)	11	(47.8)	0.25
*CDKN2A*	1	(7.1)	1	(11.1)	3	(16.7)	0.71	0	(0)	0	(0)	0	(0)	—	1	(7.7)	1	(11.1)	0	(0)	0.31
*MLH1*	0	(0)	0	(0)	0	(0)	—	0	(0)	0	(0)	0	(0)	—	0	(0)	0	(0)	0	(0)	—
*MGMT*	0	(0)	0	(0)	0	(0)	—	0	(0)	0	(0)	0	(0)	—	0	(0)	0	(0)	0	(0)	—
MINT1	10	(71.4)	8	(88.9)	14	(77.8)	0.61	1	(4.0)	1	(7.1)	1	(3.6)	0.86	1	(7.7)	2	(22.2)	0	(0)	0.08
MINT31	8	(57.1)	6	(66.7)	14	(77.8)	0.46	2	(8.0)	0	(0)	0	(0)	0.18	3	(23.1)	2	(22.2)	0	(0)	0.05
*RUNX3*	0	(0)	0	(0)	0	(0)	—	0	(0)	0	(0)	0	(0)	—	0	(0)	1	(11.1)	0	(0)	0.13
*miR-124a-3*	3	(21.4)	4	(44.4)	6	(33.3)	0.50	1	(4.0)	0	(0)	0	(0)	0.43	3	(23.1)	0	(0)	0	(0)	0.02
*miR-34c*	0	(0)	0	(0)	0	(0)	—	0	(0)	0	(0)	0	(0)	—	0	(0)	0	(0)	2	(8.7)	0.37
**No. of patients with IM**	**6**	**(%)**	**12**	**(%)**	**3**	**(%)**	***P***	**5**	**(%)**	**16**	**(%)**	**2**	**(%)**	***P***	**14**	**(%)**	**17**	**(%)**	**4**	**(%)**	***P***
MSI	3	(50.0)	5	(41.7)	0	(0)	0.32	1	(20.0)	3	(18.8)	0	(0)	0.79	3	(21.4)	2	(11.8)	0	(0)	0.51
CIMP	1	(16.7)	5	(41.7)	1	(33.3)	0.57	1	(20.0)	2	(12.5)	0	(0)	0.77	8	(57.1)	11	(64.7)	0	(0)	0.06
*CDH1*	1	(16.7)	5	(41.7)	1	(33.3)	0.57	0	(0)	2	(12.5)	0	(0)	0.62	3	(21.4)	4	(23.5)	0	(0)	0.56
*CDKN2A*	0	(0)	0	(0)	0	(0)	—	1	(20.0)	0	(0)	0	(0)	0.15	0	(0)	0	(0)	0	(0)	—
*MLH1*	0	(0)	0	(0)	0	(0)	—	0	(0)	0	(0)	0	(0)	-	0	(0)	0	(0)	0	(0)	—
*MGMT*	1	(16.7)	0	(0)	0	(0)	0.27	0	(0)	0	(0)	0	(0)	—	1	(7.1)	2	(11.8)	0	(0)	0.73
MINT1	3	(50.0)	9	(75.0)	1	(33.3)	0.32	3	(60.0)	13	(81.3)	2	(100)	0.45	11	(78.6)	14	(82.4)	1	(25.0)	0.06
MINT31	1	(16.7)	10	(83.3)	1	(33.3)	0.02	5	(100)	5	(31.3)	0	(0)	0.01	12	(85.7)	11	(64.7)	1	(25.0)	0.06
*RUNX3*	1	(16.7)	4	(33.3)	1	(33.3)	0.75	2	(40.0)	3	(18.8)	0	(0)	0.45	9	(64.3)	8	(47.1)	0	(0)	0.07
*miR-124a-3*	6	(100)	12	(100)	3	(100)	—	5	(100)	13	(81.3)	1	(50.0)	0.28	12	(85.7)	15	(88.2)	4	(100)	0.73
*miR-34c*	5	(83.3)	8	(66.7)	0	(0)	0.046	3	(60.0)	8	(50.0)	1	(50.0)	0.92	7	(50.0)	10	(58.8)	3	(75.0)	0.66

IM, intestinal metaplasia; MSI, microsatellite instability; CIMP, CpG island methylator phenotype.

*One sample in the *Hp* group and one sample in the GC group could not be analyzed due to a small sample.

In patients with IM, MINT31 and *miR-34c* methylation in the *Hp* group was significantly identified in the angulus (*P* = 0.02) and from the antrum to angulus (*P* = 0.046), respectively. Also, MINT31 methylation was the highest in the antrum (*P* = 0.01) in the AG group. In the GC group, the incidence of CIMP including MINT1, MINT31, and *runt-related transcription factor 3* (*RUNX3*) methylation, tended to be frequently found from the antrum to the angulus compared to the corpus although the number of IM samples in the corpus was very small. In addition, the methylation of *miR-124a-3* and *miR-34c* showed a high incidence throughout the stomach in each group, thus being different from the incidences in non-IM (Table 2).

The incidence of methylation in each gene and locus was shown as bar graphs in Supplementary Fig. S1. The methylation levels, which were quantified based on the melting curve by the methylation-sensitive high-resolution melting (MS-HRM) analysis, were also shown in Supplementary Fig. S2. In non-IM mucosa, methylation levels of *miR-124a-3* in the GC group showed significantly higher in the antrum (*P* = 0.008). In IM, MINT31 methylation level was significantly higher in the angulus in the *Hp* group (*P* = 0.002) and in the antrum in the AG group (*P* = 0.02), and additionally *miR-34c* methylation level showed higher in the angulus in the *Hp* group (*P* = 0.02). These results were similar to those shown by the semiquantitative assessment by MS-HRM analysis.

#### Effects of eradication in atrophic mucosa (non-IM mucosa)

The frequency of CIMP decreased significantly from the *Hp* to the AG group with eradication (odds ratio [OR]: 0.004, 95% confidence interval [CI]: 0.00025–0.075, *P* < 0.0001), but that of microsatellite instability (MSI) did not (Table 3). Although there were no significant differences in those of MSI and CIMP between the AG and GC groups, the CIMP rate tended to be higher in the GC group than in the AG group (*P* = 0.06). In contrast, *CDH1*, *CDKN2A*, MINT1, MINT31, and *miR-124a-3* methylation in non-IM mucosa were significantly decreased in the AG group compared to the *Hp* group (Table 3).

**Table 3:** Molecular alterations in non-IM among the *Hp*, AG, and GC groups.

No. of non-IM samples	*Hp* group		AG group		GC group		*Hp* vs AG group			GC vs AG group		
	n = 41	(%)	n = 67	(%)	n = 45	(%)	*P*	OR (95% CI)		*P*		
MSI	1	(2.4)	1	(1.5)	4	(8.9)	1	—		0.16		
CIMP	26	(63.4)	0	(0)	3	(6.7)	<0.0001	0.004 (0.00025–0.075)		0.06		
							**Estimate**	**SE**	***P***	**Estimate**	**SE**	***P***
*CDH1*	37	(90.2)	23	(34.3)	21	(46.7)	0.59	0.12	<0.0001	0.15	0.11	0.17
*CDKN2A*	5	(12.2)	0	(0)	2	(4.4)	0.15	0.05	0.007	0.03	0.05	0.60
*MLH1*	0	(0)	0	(0)	0	(0)	—	—	—	—	—	—
*MGMT*	0	(0)	0	(0)	0	(0)	—	—	—	—	—	—
MINT1	32	(78.0)	3	(4.5)	3	(6.7)	0.72	0.07	<0.0001	0.008	0.07	0.91
MINT31	28	(68.3)	2	(3.0)	5	(11.1)	0.63	0.08	<0.0001	0.08	0.08	0.31
*RUNX3*	0	(0)	0	(0)	1	(2.2)	<0.0001	0.02	1	0.02	0.02	0.20
*miR-124a-3*	13	(31.7)	1	(1.5)	3	(6.7)	0.32	0.07	<0.0001	0.04	0.07	0.60
*miR-34c*	0	(0)	0	(0)	2	(4.5)^a^	<0.0001	0.02	1	0.04	0.02	0.07

^a^ indicates 2 of 44.

IM, intestinal metaplasia; SE, standard error; MSI, microsatellite instability; CIMP, CpG island methylator phenotype.

#### Effects of eradication in IM

The incidences of MSI and CIMP did not show significant differences between the *Hp* and AG groups. However, the CIMP rate was significantly higher in the GC group than in the AG group (OR: 7.92, 95% CI: 1.98–31.60, *P* = 0.002), but the incidence of MSI was not (Table 4). The sensitivity, specificity, positive predictive value (PPV), and negative predictive value (NPV) of CIMP in IM for the development of GC were 54.3%, 87.0%, 86.4%, and 55.6%, respectively. The frequencies of molecular events at each gene in IM were not different between the *Hp* and AG groups, though that of *CDH1* decreased significantly after eradication (*P* = 0.01) (Table 4). When comparing genes analyzed between the GC and AG groups, MINT31 and *RUNX3* methylation tended to increase in the GC group compared to the AG group, but there were no predictive biomarkers.

**Table 4:** Molecular alterations in IM among the *Hp*, AG, and GC groups.

No. of IM samples	*Hp* group		AG group		GC group		*Hp* vs AG group			GC vs AG group		
	n = 21	(%)	n = 23	(%)	n = 35	(%)	*P*			*P*	OR (95% CI)	
MSI	8	(38.1)	4	(17.4)	5	(14.3)	0.18			1		
CIMP	7	(33.3)	3	(13.0)	19	(54.3)	0.16			0.002	7.92 (1.98–31.60)	
							**Estimate**	**SE**	***P***	**Estimate**	**SE**	***P***
*CDH1*	7	(33.3)	2	(8.7)	7	(20.0)	0.33	0.13	0.01	0.11	0.12	0.35
*CDKN2A*	0	(0)	1	(4.3)	0	(0)	−0.03	0.03	0.26	−0.03	0.02	0.20
*MLH1*	0	(0)	0	(0)	0	(0)	—	—	—	—	—	—
*MGMT*	0	(0)	0	(0)	3	(8.6)	0.04	0.07	0.56	0.10	0.06	0.11
MINT1	13	(61.9)	18	(78.3)	26	(74.3)	−0.15	0.15	0.30	−0.02	0.13	0.85
MINT31	12	(57.1)	10	(43.5)	24	(68.6)	0.19	0.16	0.24	0.27	0.14	0.07
*RUNX3*	6	(28.6)	5	(21.7)	17	(48.6)	0.05	0.14	0.71	0.23	0.12	0.07
*miR-124a-3*	21	(100)	19	(86.4)^a^	31	(88.6)	0.13	0.11	0.24	−0.02	0.09	0.85
*miR-34c*	13	(61.9)	12	(54.5)^b^	20	(58.8)^c^	−0.01	0.17	0.95	−0.01	0.15	0.92

^a,b^ and ^c^ indicate 19 of 22, 12 of 22, and 20 of 34, respectively.

IM, intestinal metaplasia; SE, standard error; MSI, microsatellite instability; CIMP, CpG island methylator phenotype.

### Comparison of paired non-IM and IM from individual patients

The molecular events were compared between paired non-IM mucosa and IM in all samples, including the *Hp*, AG, and GC groups, by conditional logistic regression models. After adjusting in the same patients, MSI, CIMP, *CDH1*, MINT1, MINT31, *RUNX3*, and *miR-34c* methylation were specific markers that were detected in IM compared to non-IM mucosa (Table 5).

**Table 5:** Comparison of IM versus non-IM in all samples adjusted for the same patients.

Variable	IM vs non-IM		
	Odds ratio	95% confidence interval	*P*
MSI	5.90	1.63–21.35	0.007
CIMP	4.45	1.44–13.78	0.001
*CDH1*	0.16	0.06–0.42	0.0002
*CDKN2A*	0.25	0.02–2.76	0.26
*MLH1*	—	—	—
*MGMT*	—	—	—
MINT1	13.74	4.84–39.06	<0.0001
MINT31	5.96	2.55–13.93	<0.0001
*RUNX3*	22.92	3.02–173.75	0.002
*miR-124a-3*	—	—	—
*miR-34c*	43.69	5.91–322.84	0.0002

IM, intestinal metaplasia.

### Changes over time in molecular events after *H. pylori* treatment

#### Molecular changes in non-IM mucosa

**Figure 1:**
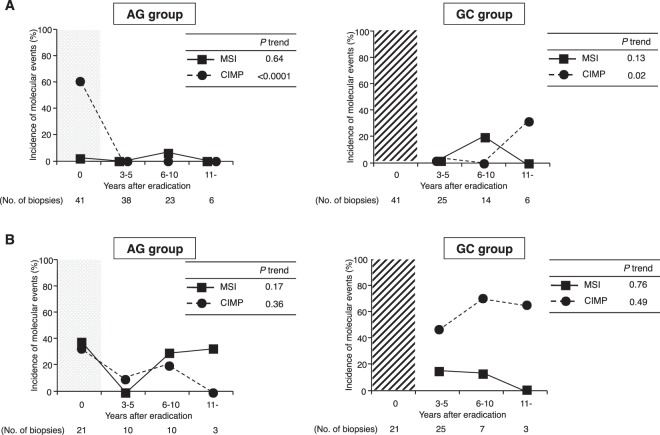
Changes of MSI and CIMP in the AG and GC groups. (**A**) The data points within the shaded t = 0 region indicate the *Hp* group. In non-IM, both MSI and CIMP have mostly disappeared after *H*. *pylori* eradication in the AG group. The incidence of CIMP increases gradually, but does not show a significant change in the GC group. (**B**) In IM, the incidence of MSI and CIMP do not show significant changes after treatment in the AG group. The incidence of CIMP remains persistently high in the GC group.

**Figure 2:**
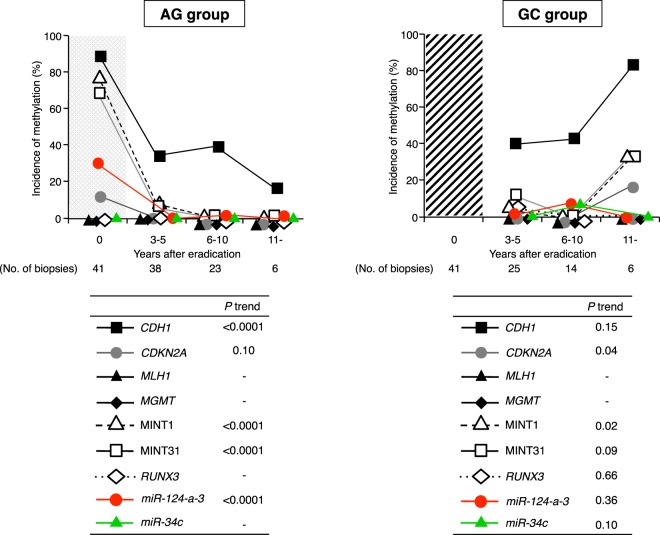
Changes of molecular alterations in non-IM after *H*. *pylori* eradication in the AG and GC groups. The data points within the shaded t = 0 region indicate the *Hp* group. In non-IM, methylation of most genes evaluated disappears after eradication. Although the incidence of *CDH1* gene methylation decreases over time, the methylation does not disappear completely for up to more than 11 years. In the GC group, the incidence of *CDH1*, *CDKN2A* (*P* trend = 0.04), MINT1 (*P* trend = 0.02), and MINT31 gene methylation increases gradually after 11 years of eradication. *miR-124a-3* methylation and *miR-34c* methylation are minimally detected after *H*. *pylori* treatment in both the AG and GC groups.

MSI and CIMP disappeared after eradication, and the conditions persisted for more than 11 years in the AG group, while the incidence of CIMP increased gradually with time in the GC group (*P* trend = 0.02) (Fig. 1A). Although the incidence of *CDH1* methylation decreased with time in the AG group, the methylation did not completely disappear up to more than 11 years (Fig. 2). In the GC group, *CDH1* methylation increased persistently from 3 to 11 years, and methylation at the MINT1 and MINT31 loci and *CDKN2A* gene increased after 11 years of eradication in the same manner as at the *CDH1* gene (Fig. 2). In contrast, there was very little *miR-34c* methylation, and *miR-124a-3* was minimally detected after *H*. *pylori* treatment in both groups.

#### Molecular changes in IM

**Figure 3:**
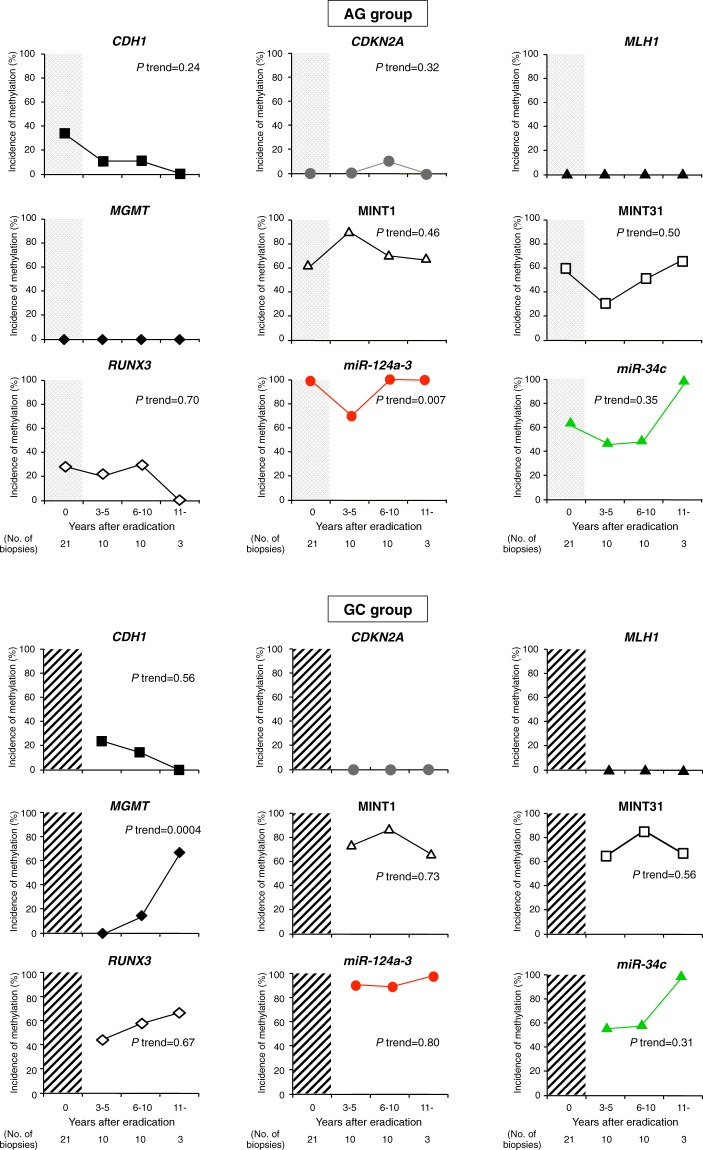
Changes of molecular alterations in IM after *H*. *pylori* eradication in the AG and GC groups. In IM, MINT1 methylation and MINT31 methylation maintain a high incidence, and methylation of other genes does not also show a significant change in the AG group. In the GC group, the incidences of MINT1, MINT31, and *RUNX3* methylation remain persistently high, and that of *MGMT* methylation gradually increases more than 11 years after eradication (*P* trend = 0.0004). *miR-124a-3* methylation and *miR-34c* methylation in IM are persistently high even after eradication in both groups.

In the AG group, the incidences of MSI and CIMP did not show significant changes after eradication in the AG group. In the GC group, the incidence of CIMP remained persistently high (Fig. 1B). The methylation of MINT1 and MINT31 remained high, and methylation of the other genes did not show a significant change even after eradication in the AG group (Fig. 3). In the GC group, the incidence of MINT1, MINT31, and *RUNX3* methylation also remained persistently high, and that of *O6-methylguanine-DNA methyltransferase* (*MGMT*) methylation increased gradually at more than 11 years after eradication (*P* trend = 0.0004) (Fig. 3). Methylation of *miR-124a-3* and *miR-34c* in IM was persistently high in both groups even after *H*. *pylori* eradication.

### mAb Das-1 reactivity and E-cadherin immunostaining

**Figure 4:**
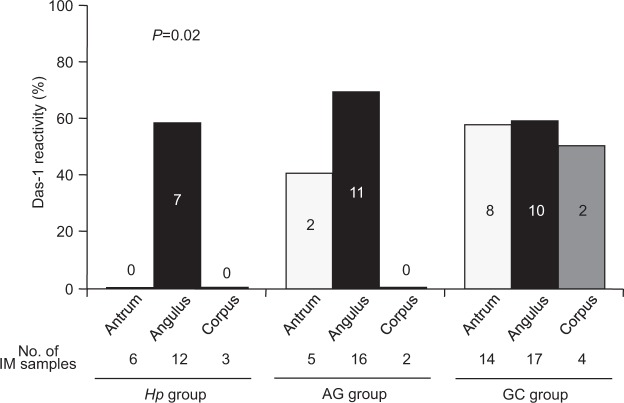
mAb Das-1 reactivity to IM in different parts of the stomach in the three groups. The reactivity is the highest in the angulus compared to the other parts in each group, especially in the *Hp* group (*P* = 0.02). In the GC group, mAb Das-1 reactivity is high throughout the stomach, and thus there is a significant difference in reactivity in the antrum and corpus sites between the *Hp* (0%, 0 of 9) and GC groups (55.6%, 10 of 18) (*P* = 0.009).

Figure 4 shows the mAb Das-1 reactivity against IM in three parts of the stomach in each group. The reactivity was highest in the angulus compared to the other parts of the stomach in the *Hp* (*P* = 0.02) and AG groups. Interestingly, in the GC group, mAb Das-1 reactivity was high throughout the stomach.

When evaluating the relationship between E-cadherin immunostaining and *CDH1* methylation, there was no significant correlation between altered expression and *CDH1* methylation. In addition, the sensitivity, specificity, PPV, and NPV of altered E-cadherin expression in non-IM and IM with methylated *CDH1* gene were 40.7%, 66.7%, 57.9%, 50.0% and 43.8%, 60.3%, 21.9%, 80.9%, respectively (Supplementary Table S1).

## Discussion

This is the first study to show the long-term effects of *H*. *pylori* eradication on molecular alterations in precancerous mucosa, e.g. atrophic mucosa (non-IM) and IM, with and without early GC. In this study, *H*. *pylori* eradication was associated with a significant reduction of CIMP, including *CDH1*, *CDKN2A*, MINT1, MINT31, and *miR-124a-3* methylation, in non-IM mucosa but not in IM. In addition, CIMP in IM may be a surrogate marker of GC risk. Interestingly, *miR-124a-3* methylation and *miR-34c* methylation are markers of dysregulation of noncoding RNA that occurred in IM but not in non-IM, and thus may not necessarily be associated with GC risk, unlike in previous reports^24,26–29^.

*H*. *pylori* infection causes aberrant DNA hypermethylation of specific genes and induces CIMP, which is an important epigenetic mechanism of tumorigenesis^35,36^. The accumulation of aberrant DNA methylation in non-cancerous tissues was recognized as the “epigenetic field for cancerization”, especially in inflammation-associated cancers such as GC^36–39^. Although several studies have demonstrated changes in DNA methylation after *H*. *pylori* eradication^14–16,18,19^, no study has examined long-term aberrant methylation status after treatment, especially taking into consideration the presence or absence of IM in the background mucosa. In this study, the frequency of CIMP in non-IM, which shows widespread CpG island methylation including various genes/loci such as *CDH1*, *CDKN2A*, MINT1, and MINT31 methylation, decreased significantly with *H*. *pylori* eradication. Previous studies have shown that *CDH1* methylation is strongly associated with *H*. *pylori* infection^11,14–16^, and it has been frequently observed in precancerous lesions^11,15,16^. In the present result, the *CDH1* methylation rate decreased gradually over time after *H*. *pylori* eradication in both non-IM and IM. Chan *et al*. also demonstrated that methylation of the *E-cadherin* (*CDH1*) promoter reversed after *H*. *pylori* eradication, and the disappearance of *E-cadherin* methylation may be important for preventing the future development of GC^15^. However, the follow-up period in their study was very short (6 weeks), and they did not separately evaluate the methylation status in non-IM and IM using the laser capture microdissection (LCM) system. Furthermore, methylation-specific PCR (MSP), which was used for the methylation analysis, has some limitations; it gives many false-positive results and is qualitative^40^. Interestingly, the frequencies of methylation of *CDH1*, *CDKN2A* (*P* trend = 0.04), and MINT1 (*P* trend = 0.02) in non-IM mucosa increased in the GC group (Fig. 2). In addition, the incidence of only *MGMT* methylation in IM increased gradually with time after eradication in the GC group (*P* trend = 0.0004) (Fig. 3). A recent meta-analysis showed that *MGMT* gene hypermethylation was significantly associated with an increased risk of GC, especially in Asians^41^. Therefore, a long-term follow-up study with a larger sample size will be needed to confirm more clearly the relationship between the changes of methylation of these genes following *H*. *pylori* eradication and GC development.

In the present study, the incidence of molecular alterations related to carcinogenesis was significantly higher in IM than in non-IM, irrespective of the presence or absence of *H*. *pylori* infection and GC in the background mucosa (Table 5), and *H*. *pylori* eradication was less effective in reversing the methylation that occurred in IM than in non-IM. Therefore, these results indicate the concept of the “point of no return”^42^, in which the benefits of *H*. *pylori* eradication diminish after the development of IM accompanied with molecular changes. More recently, we reported that the long-term use of aspirin for more than 3 years decreases *CDH1* methylation in non-IM and CIMP in IM in patients with chronic gastritis who regularly took aspirin for more than 3 years, but was not very effective in reversing the methylation that occurred in IM^43^. In the present study, *H*. *pylori* eradication significantly reduced the methylation of not only *CDH1* but also *CDKN2A*, MINT1, MINT31, and *miR-124a-3* in non-IM, and of *CDH1* in IM, whereas the eradication did not decrease CIMP in IM. The results of this study, which analyzed patients that were not taking aspirin, and with a cohort that was distinct from the previous study^43^, were different from those of the previous study analyzing the effects of aspirin^43^. Meanwhile, *H*. *pylori* eradication was similarly less effective in improving the methylation in IM compared with non-IM; thus being similar to the previous results of the effects of aspirin use^43^. Taking into account the present study and our previous study^43^, *H*. *pylori* eradication is recommended for patients with chronic atrophic gastritis at an early stage prior to the development of IM to prevent GC development. Additionally, long-term aspirin use, as an additional chemopreventive therapy, may be beneficial to avoid GC development in patients with IM.

It has been reported that *H*. *pylori* infection can induce DNA methylation of miRNA genes^24,27–29^, and several tumor-suppressor miRNAs, including *miR-124a* and *miR-34b/c*, were silenced by DNA hypermethylation of their promoter CpG islands in GC^24,26–29^. According to the recent studies, *miR-124a-3* methylation in the background mucosa was associated with an increased risk of developing metachronous GCs^24,27^. Similarly, methylation of *miR-34b/c* is also reported to be a predictive marker of GC risk, especially multiple and metachronous GCs^28,29^. However, the present study found that the incidences of methylation of *miR-124a-3* and *miR-34c* were not different among the three groups except in non-IM from the *Hp* group, and they were mostly observed in IM, with very few in non-IM. Thus, these results indicate that the methylation of these miRNA genes might be a specific marker expressed in IM and might not necessarily be a risk marker for GC, which was not in agreement with the previous reports^24,27–29^. One of the explanations for this discrepancy may be the difference in the DNA extraction method. In the previous studies, whole biopsy tissues were used for DNA methylation analysis, and the differences in methylation between non-IM and IM were not evaluated. GC risk is generally considered to relate directly to the extent of IM^44^. Therefore, the results^24,27–29^ may be affected by the amount of IM glands contained in the biopsy samples. On the other hand, the reason why the frequency of *miR-124a-3* methylation was relatively high in non-IM in the *Hp* group may be influenced by the contamination of inflammatory cells in the biopsy samples because DNA from non-IM samples were extracted from the whole biopsy specimens.

In the present study, biopsies were taken from three parts of the stomach. When the frequencies of molecular alterations were compared among the sites, MINT31 and *miR-124a-3* methylation was significantly detected in the antrum to the angulus in patients with non-IM from the GC group although no significant difference was detected in the frequency among the three sites in the *Hp* and AG group. Additionally, molecular events such as MINT1, MINT31, and *RUNX3* methylation including CIMP were frequently identified from the antrum to the angulus in patients with IM, especially in the GC group. As the number of IM in the corpus was very small in each group, the accurate molecular anomalies may not be reflected in the results. However, these results may support the clinical fact that *H*. *pylori*-associated GC is related to the extent of IM and mainly develops in the distal portion of the stomach, i.e., from the antrum to the angulus^44^. Also, the result in which methylation status extensively spreads in the stomach may strongly suggest the concept of the “epigenetic field for cancerization”^36–39^, especially in the GC group. A recent study by Huang *et al*. showed that methylation patterns were distinctly different between the gastric antrum and body or cardia^45^, which was in agreement with the present study. The incidence of mAb Das-1 reactivity was the highest in the angulus of the stomach, and the reactivity in the GC group was high throughout the stomach. There was no significant difference in mAb Das-1 reactivity against IM among the three groups; 33.3% (7 of 21) in the *Hp* group, 56.5% (13 od 23) in the AG group, and 57.1% (20 of 35) in the GC group. In our previous studies^22,34^, however, the reactivity was significantly higher in the GC group than in the *Hp* group. The reason for the discrepancy might be that reactivity was analyzed with two biopsy specimens taken from the greater curvature of the antrum and corpus of the stomach^22,34^. When evaluating the two biopsy samples (antrum and corpus) in the present study, mAb Das-1 reactivity was significantly higher in the GC group (10 of 18, 55.6%) than in the *Hp* group (0 of 9, 0%) (*P* = 0.009) (Fig. 4), as in our previous studies^22,34^. However, the reason why mAb Das-1 reactivity was consistently high in the angulus among the three groups remains unclear.

The present results showed that altered expression of E-cadherin was not correlated with *CDH1* methylation, which has low sensitivity, specificity, PPV, and NPV, thus being similar to the results of the studies by Chan *et al*.^11,15^. They mentioned that immunohistochemical staining is a qualitative method and is not as sensitive as PCR in detecting subpopulations of cells with gene methylations^15^. However, since they analyzed methylation status using MSP, unlike the present method, our present results may show a stronger tendency for a lack of concordance between altered E-cadherin expression and *CDH1* gene methylation.

The MS-HRM used in our study is applicable for semiquantitative assessment of methylation levels in an unmethylated background. This method allows for the efficient screening of a sample to rapidly detect the methylation level and is more cost-effective than pyrosequencing, which counts every CpG site and requires a specialized instrument. In this study, we attempted to quantify the melting curve obtained by the MS-HRM method. Although the correlation coefficient of the calibration curve from the fluorescence value of the melting curve using the methylation standard control DNA was very high (Supplementary Fig. S3), the data may lack objectivity and accuracy because we performed the conversion manually. However, when comparing between the incidence of methylation by the MS-HRM (cut-off >10%) and calculating median values of methylation levels in each gene, the differences of the methylation rates or methylation levels in both non-IM and IM among the three parts of the stomach showed completely analogous patterns in each group, thus the definition of methylation (>10%) by MS-HRM may be reasonable.

The present study has some limitations. First, this was a study from a single institution with a small number of patients. Second, the number of cases followed-up for a long term (>10 years) was small, particularly considering that several different molecular markers and cellular phenotypes were compared. Third, this was a cross-sectional study, which inevitably includes various types of biases. However, consecutive patients with chronic AG and early GC who were followed-up for at least more than 3 years after *H*. *pylori* eradication were included. Fourth, in order to clarify long-term molecular biological effects of *H*. *pylori* eradication, sequential biopsy samples in the same patients are necessary for time-course analysis. However, a sequential biopsy in the same patients was not performed in the present study because biopsies were performed only once in each group. In patients from the AG and GC groups, biopsies were taken at various times (≥3 years) after *H*. *pylori* eradication; these results were a substitute for a time-course analysis. Fifth, we did not analyze the effects of proton pump inhibitors (PPIs) in this study. Recently, the association between long-term use of PPIs and GC risk in patients after *H*. *pylori* eradication has been discussed^46,47^. In our study, PPI users comprised 47.6% (10 of 21 subjects) of the *Hp* group, 33.3% (10 of 30 subjects) of the AG group, and 37.0% (10 of 27 subjects) of the GC group, and there was no statistically significant difference between the groups (*P* = 0.58). Therefore, there may not be any direct or indirect effects by PPIs in the present study. Further studies with a larger sample size are needed to clarify this association using molecular pathological analyses.

In conclusion, in cases of patients with long-term follow-up after *H*. *pylori* eradication: (1) *H*. *pylori* eradication was associated with a significant reduction of CIMP in non-IM, but not in IM; (2) CIMP in IM may be a surrogate marker of GC; and (3) *miR-124a-3* methylation and *miR-34c* methylation are molecular events that occur specifically in IM, and they may not be associated with GC development.

## Patients, Materials and Methods

### Patients

A cross-sectional study was conducted of patients with early GC who underwent ER between March 2011 and December 2016 at Hyogo College of Medicine Hospital (Hyogo, Japan). During this period, 522 patients with a total of 615 dysplasias comprising gastric adenomas (n = 50) and other GCs (n = 565) were treated endoscopically. Among them, 44 patients (7.8%) who had undergone *H*. *pylori* eradication more than 3 years earlier had developed primary gastric dysplasia but not metachronous GC. However, because informed consent was not obtained from 17 of the patients, the following were finally enrolled: patients who developed primary early GC despite the successful eradication of *H*. *pylori* more than 3 years before (GC group, n = 27); patients with histologically AG (GC-free patients) who had undergone *H*. *pylori* eradication more than 3 years before and had AG in the background mucosa (AG group, n = 30); and patients with histologically AGs (GC-free patients) who were positive for *H*. *pylori* (*Hp* group, n = 21). Patients from the *Hp* and AG groups were randomly selected during the same period. Histological diagnosis of GC was made in accordance with the criteria of the Japanese Research Society for Gastric Cancer^48^. Patients with a history of esophagectomy or gastrectomy and taking aspirin or other nonsteroidal anti-inflammatory drugs were excluded.

### Consent and institutional review board approval

Written, informed consent was obtained from all patients prior to this study. The Ethics Committee of Hyogo College of Medicine approved this study (Nos. Rin-Hi 136 and 300). This trial was registered with the UMIN Clinical Trials Registry (No. UMIN000021857). The study was performed in accordance with the Declaration of Helsinki.

### *H. pylori* status and DNA extraction

During each patient’s endoscopy, three biopsy specimens were taken from three parts of the stomach, the greater curvatures of the antrum and corpus, and the lesser curvatures of the angulus (one from each site). Biopsy was done only once in each group at the time when the above conditions were satisfied. In the GC group, biopsies were taken from the background mucosa immediately after ER for early GC. Each biopsy specimen, which was cut into 4-µm-thick tissue sections, was used for histological analysis by hematoxylin and eosin staining, Giemsa staining, and mAb Das-1 and E-cadherin staining. *H*. *pylori* status was analyzed in each patient by the following methods: the urea breath test (UBT), Giemsa staining, and the E-plate anti-*H pylori* IgG antibody test (Eiken Kagaku, Tokyo, Japan). Before eradication, a patient was regarded as *H*. *pylori-*positive if the result of at least one of the three aforementioned methods was positive. *H*. *pylori* status following the eradication was determined by the UBT at least 6 weeks or more after the end of anti-*H*. *pylori* treatment. From the paraffin-embedded biopsy specimens, furthermore, two or three 7-µm-thick tissue sections were cut for DNA extraction. DNA was extracted from goblet IM (incomplete type) using the QIAamp DNA Micro Kit (Qiagen, Hilden, Germany). At least ten goblet IM glands were isolated using the PALM MicroBeam LCM system (Microlaser Technologies, Munich, Germany)^22,25,43^ (Supplementary Fig. S4). In contrast, when IM glands were not identified in the biopsy sample, DNA was extracted from the whole biopsy tissue as DNA from non-IM mucosa. One sample obtained from the angulus in the *Hp* group and one sample from the corpus in the GC group could not be analyzed for molecular alterations due to the small amount of DNA that was extracted from the very small biopsy specimen. Finally, a total of 232 biopsy samples from 78 patients were analyzed. In this study, molecular events and characteristics, including MSI, methylation of CpG islands of various genes, CIMP, mAb Das-1 reactivity, and E-cadherin overexpression were analyzed.

### Analysis of MSI by high-resolution fluorescent microsatellite analysis

As previously reported^22,25,43,49^, the following five microsatellite loci on chromosomes were examined for MSI based on the revised Bethesda panel^48–50^: BAT26, BAT25, D2S123, D5S346, and D17S250. MSI status was judged according to previous reports^43,49,51,52^ (Supplementary Fig. S5). In cases in which MSI and loss of heterozygosity were indistinguishable^52^, the allelic imbalance (AI) ratio was calculated. MSI was determined to be positive when the AI ratio (normal allele 1:normal allele 2/tumor allele 1:tumor allele 2) was <0.67 or >1.35, as previously reported^43,49,51^. The lesions were defined as MSI when unstable loci were observed in two or more of the five investigated markers^43,49^.

### Sodium bisulfite modification of DNA and CIMP markers

Similar to previous reports^25,43,49^, purified DNA samples were chemically modified by sodium bisulfite with an EpiTect^®^ Fast Bisulfite Kit (Qiagen). The bisulfite-modified DNA was amplified using primer pairs that specifically amplify the methylated or unmethylated sequences of several genes/loci related to carcinogenesis, including *CDH1*, *CDKN2A* (*p16*), *MLH1*, MINT1, MINT31, *MGMT*, and *RUNX3*. These genes were used as CIMP markers. A recent study found marked variability in the genes used in the selection panel for determining gastric CIMP status^53^. Although there are two major CIMP panels, i.e., the classic panel^54^ and the novel marker panel^55^, there is also no gold standard with respect to gene panels and the number of marker thresholds used to define CIMP even in colorectal CIMP^56^. CIMP status generally implies methylation in at least two MINTs or target genes such as *p14*, *p16*, or *MLH1* when a small panel of markers is needed^57,58^. Therefore, CIMP was analyzed using the above seven panels based on our previous report^43,49^. Additionally, methylation of *miR-124a-3* and *miR-34c*, which are associated with GC risk^17,24,26,28,29^, was also evaluated. However, among the samples for analysis of these miRNAs, one from each *miR-124a-3* methylation and *miR-34c* methylation in IM in the AG group and one from *miR-34c* methylation in IM in the GC group could not be analyzed due to the small amount of DNA.

### Methylation-sensitive high-resolution melting analyses

Methylation-sensitive high-resolution melting (MS-HRM) analysis was performed as we previously described^25,43,49^. Briefly, PCR amplification and MS-HRM analysis were performed using a LightCycler^®^ 480 System II (Roche, Mannheim, Germany). PCR amplification of the converted DNA was carried out in a 20-μL reaction volume containing 10 μL of 2× Master Mix (LightCycler^®^ 480 High Resolution Melting Master, Roche), 0.5 μL of each primer (10 pmol/μL), 2.4 μL of MgCl_2_, 1.6 μL RNase-free water, and 10 ng of bisulfite-modified template DNA following the manufacture’s protocol (Roche). The primer sequences of all genes for the methylated and unmethylated forms and PCR and MS-HRM conditions are summarized in Supplementary Tables S3 and S4. Percentages of methylation (0%, 10%, 50%, and 100%) were used to draw the standard curve (Supplementary Fig. S6). In this study, only samples with >10% methylation were considered methylated^25,43,49^. CIMP was defined as ≥3/7 methylated markers using the seven-marker CIMP panel^43,49^.

### An attempt to quantify the melting curve obtained by the MS-HRM analysis

We prepared a calibration curve from the fluorescence value of the melting curve using the methylation standard control DNA (0%, 10%, 50%, and 100% Methylated), and estimated the methylation levels of individual genes (Supplementary Fig. S3). The results showed that the correlation coefficient of the calibration curve showed very high (*r*^2^ = 0.998).

### Immunohistochemical staining of mAb Das-1 and E-cadherin

Serial sections were stained with mAb Das-1 (a highly specific IgM mAb against the colonic phenotype) using sensitive immunoperoxidase assays, as previously described^22,25,33,34^. mAb Das-1 does not react with normal gastric mucosa and non-IM other than IM^22,25,33,34^. Consequently, positive expression was defined as greater than 10% of IM glands stained with mAb Das-1^43^ (Supplementary Fig. S7A). The streptavidin-biotin-peroxidase complex method was used for the detection of a mouse mAb against E-cadherin (E-cadherin (G-10), Santa Cruz Biotechnology, Inc., Dallas, TX) according to our previous report^59^ (Supplementary Fig. S7B,C). E-cadherin staining was judged as altered expression if reduced expression in the plasma membrane and/or cytoplasmic localization of E-cadherin was identified in more than 20% of the gastric mucosa^59^.

The diagram illustrating the origin and the fate of the biopsies mentioned above was provided in the Supplementary Fig. S8.

### Statistical analysis

Continuous and categorical data are reported as means and standard deviations (SDs) and medians (1^st^–3^rd^ quartile) and frequencies with proportions, respectively. The data were assessed by the Mann-Whitney *U-*test for comparisons between two independent groups, by the Kruskal-Wallis test for comparisons among the three independent groups, and by the chi-squared test or Fisher’s exact test for comparisons of proportions. General linear regression models were used to evaluate the differences of molecular alterations between the *Hp* and AG groups and between the GC and AG groups in non-IM and IM. Conditional logistic regression analysis was performed to compare the molecular events of paired non-IM and IM from individual patients and to estimate the odds ratio with 95% confidence interval. A two-tailed *P*-value less than 0.05 was considered significant. Statistical analyses were performed with SPSS 22.0 (SPSS Inc., Chicago, IL) and SAS 9.4 (SAS Institute Inc., Cary, NC).

## Electronic supplementary material

Supplementary Figures and Tables
