# Supplementary material for: Long-term effects of H. pylori eradication on epigenetic alterations related to gastric carcinogenesis
Source: Sci Rep. 2018 Sep 25;8:14369. doi: 10.1038/s41598-018-32717-3 (PMC6156585; doi:10.1038/s41598-018-32717-3)
Supplement: Supplementary file 1 — Supplementary Figures and Tables [file 41598_2018_32717_MOESM1_ESM.docx]

**Long-term effects of *H. pylori* eradication on epigenetic alterations related to gastric carcinogenesis**

Yuki Michigami ^1^, Jiro Watari ^1^, Chiyomi Ito ^1^, Keisuke Nakai ^1^, Takahisa Yamasaki ^1^, Takashi Kondo ^1^, Tomoaki Kono ^1^, Katsuyuki Tozawa ^1^, Toshihiko Tomita ^1^, Tadayuki Oshima ^1^, Hirokazu Fukui ^1^, Takeshi Morimoto ^2^, Kiron M. Das ^3^, Hiroto Miwa ^1^

^1^ Division of Gastroenterology, Department of Internal Medicine, Hyogo College of Medicine, Nishinomiya, Japan; ^2^ Department of Clinical Epidemiology, Hyogo College of Medicine, Nishinomiya, Japan; ^3^ Division of Gastroenterology and Hepatology, Departments of Medicine and Pathology, Robert Wood Johnson Medical School, Rutgers, Cancer Institute of New Jersey, New Brunswick, United States

**Supplementary Figure Legends**

**Supplementary Figure S1.** Incidence of methylation in the three portions of the stomach among the three groups using semiquantitative analysis by the MS-HRM (cut-off >10% for methylation). (A) In non-AM, *miR-124a-3* methylation is more frequently found in the antrum than in the other parts in the GC group (*P*=0.02). (B) In IM, the incidence of MINT31 methylation is significantly higher in the angulus in the *Hp* group (*P*=0.02) and in the antrum in the AG group (*P*=0.01) compared to the other parts. *miR-34c* methylation is more frequently identified in the antrum to the angulus than in the corpus in the *Hp* group (*P*=0.046).

**Supplementary Figure S2.** Methylation levels in the three parts of the stomach among the three groups. A horizontal line represents the median value for each group. Statistical analyses where performed using the Kruskal-Wallis test. (A) In non-IM, the methylation level of *miR-124a-3* is significantly higher in the antrum than in the other parts in the GC group (*P*=0.008). (B) In IM, the methylation level of MINT31 is significantly higher in the angulus in the *Hp* group (*P*=0.002) and in the antrum in the AG group (*P*=0.02). In addition, the methylation level of *miR-34c* is higher in the antrum and angulus than in the corpus in the *Hp* group (*P*=0.02). Taking into consideration these results from Supplementary Figure S1 and S2, with respect to the differences of the methylation rate in the three parts of the stomach, semiquantitative analysis by the MS-HRM (cut-off >10% for methylation) is the same as calculated by quantitative analysis.

**Supplementary Figure S3.** (A) Melting curve of *RUNX3* gene by MS-HRM analysis. (B) Correlation between the methylation level of *RUNX3* and fluorescence intensity when calculated from the melting curve. The correlation coefficient of the calibration curve from the fluorescence value of the melting curve using the methylation standard control DNA is very high.

**Supplementary Figure S4.** (A) The glands of intestinal metaplasia (IM) were isolated by laser capture microdissection. (B) The same section after the removal of IM glands.

**Supplementary Figure S5.** Examples of MSI detected in non-IM by high-resolution fluorescent microsatellite analysis. (A) MSI on D2S123 is identified by the appearance of multiple additional peaks (arrows). (B) MSI on BAT25 is seen as an unequivocal extra peak shift (asterisk) compared with control. (C) T1 and N1 represent the highest respective peak areas of the shorter allele in non-IM, and control samples and T2 and N2 represent the highest respective peak areas of the longer allele. MSI on D2S123 (longer allele, T2) of non-IM DNA is seen. The allelic imbalance ratio is 0.31.

**Supplementary Figure S6.** Representative results of MS-HRM analysis for methylation. Results are shown for the *miR-124a-3* gene, with positive (fully methylated) in IM in the GC group and negative controls (fully unmethylated). The melting peaks were calculated from melting curves of MS-HRM. Each sample was directly compared with its control to identify the sample’s methylation status, and the differences in fluorescence between samples were normalized by the analysis algorithms. Methylated and partially methylated DNA (≥ 10%) were considered to be positive for methylation, and unmethylated DNA was treated as negative. The sample shows a moderate level of methylation (≥ 50%).

**Supplementary Figure S7.** Representative section of immunohistochemistry with mAb Das-1 and E-cadherin. (A) Das-1 reactivity in the IM is demonstrated in the GC group (X200). (B) Positive staining with E-cadherin antibody is found at the surfaces of atrophic mucosa (X200), whereas this biopsy sample shows *CDH1* methylation. (C) Although IM glands are positive for E-cadherin (X200), CpG island methylation at the *CDH1* gene is identified in these glands.

**Supplementary Figure S8.** A diagram illustrating the origin and fate of the biopsies.

**Supplementary Figure S8**

**Supplementary Table S1.** Relationship between E-cadherin immunostaining and *CDH1* methylation in non-IM and IM

1. Non-IM

|  | *CDH1* gene | |  |
| --- | --- | --- | --- |
| E-cadherin immunostaining | Methylated | Unmethylated | *P* |
| Altered | 33 | 24 | 0.34 |
| Positive | 48 | 48 |  |

Statistical calculations for altered E-cadherin expression in non-IM with methylated *CDH1* gene: sensitivity 40.7%, specificity 66.7%, positive predictive value 57.9%, and negative predictive value 50.0%

2. IM

|  | *CDH1* gene | |  |
| --- | --- | --- | --- |
| E-cadherin immunostaining | Methylated | Unmethylated | *P* |
| Altered | 7 | 25 | 0.77 |
| Positive | 9 | 38 |  |

Statistical calculations for altered E-cadherin expression in IM with methylated *CDH1* gene: sensitivity 43.8%, specificity 60.3%, positive predictive value 21.9%, and negative predictive value 80.9%

IM, intestinal metaplasia

Supplementary Table S3. Primer sequences for the MS-HRM assays

| Gene/Locus | Primer sequences (5’ – 3’) |
| --- | --- |
| *CDH1* | F - GGAATTGTAAAGTATTTGTGAGTTTG |
|  | R - AAAATACCTTCAACCAATCACCTC |
| *CDKN2A* | F- CGGAGGAAGAAAGAGGAGGGGT |
|  | R- CGCTACCTACTCTCCCCCTCT |
| *MLH1* | F - TTGGTATTTAAGTTGTTTAATTAATAGTTG |
|  | R - AAAATACCTTCAACCAATCACCTC |
| *MGMT* | F- GTTTCGGGTTTCGTATTTATTTTGAAGG |
|  | R- GACAACCCCAACTTCCTCTACTC |
| MINT1 | F- GGGGTTGAGGTTTTTTGTTAG |
|  | R- AATCCCTCTCCCCTCTAAACTT |
| MINT31 | F- GGGTGATGGTTTTAGTAAAGTGAG |
|  | R- AAAAACACTTCCCCAACATCTAC |
| *RUNX3* | F- GTTTCGGGTTTCGTATTTATTTTGAAGG |
|  | R- GACAACCCCAACTTCCTCTACTC |
| *miR-124a-3* | F- GGGAGAAGTGTGGGTTTTTT |
|  | R- CCTTAATTATATAAACATTAAATCAAAATC |
| *miR-34c* | F- GATTGTATTGTGGTGGTTATAATTATTAAT |
|  | R- CCTCCAAAAATTTTACTTTCCTAAC |

**Supplementary Table S4.** PCR and MS-HRM conditions in each gene

| Pre-incubation | | |  |  | Amplification | | | | | |  |  | Cooling | | |
| --- | --- | --- | --- | --- | --- | --- | --- | --- | --- | --- | --- | --- | --- | --- | --- |
| Denaturation (°C) | Hold  (min) | Ramp rate  (°C/s) |  |  | Target  (°C) | Hold  (sec) | Ramp rate  (°C/s) | Sec target^*^  (°C) | Step size^*^  (°C) | Step delay^*^  (°C) | Cycles |  | Target  (°C) | Hold  (sec) | Ramp rate  (°C/s) |
| 95 | 10 ^a,b,c,d,e,f^  15 ^g^ | 4.8 |  | Denaturation | 95 | 10 ^c,d,e,f^  15 ^g,h,i^  30 ^a,b^ | 4.8 |  |  |  | 45 ^a,b,d,e^  50 ^c,f,g^  52 ^h,i^ |  | 40 | 30 | 2.5 |
|  |  |  |  | Annealing | 51 ^d^, 55 ^g^  59 ^h^, 60 ^i^  62 ^e^, 63 ^b^  64 ^c^, 65 ^a,f^ | 10 ^d,e^  15 ^g^  30 ^a,b,c,f,h,i^ | 2.5 | 50 ^e^, 52 ^c^  53 ^f^, 54 ^h^  55 ^i^, 57 ^b^  60 ^a^ | 0.5 ^a,b,c,e,f,h,i^ | 1 ^a,b,c,e,f,h,i^ |  |  |  |  |  |
|  |  |  |  | Extension | 72 | 10 ^d,e^  15 ^b^  20 ^g^  30 ^a,c,f,h,i^ | 4.8 |  |  |  |  |  |  |  |  |
|  |  |  |  |  |  |  |  |  |  |  |  |  |  |  |  |
|  |  |  |  |  | High-resolution melting | | | | | |  |  |  |  |  |
|  |  |  |  | Denaturation | 95 ^a,b,c,d,e,f,h,i^  97 ^g^ | 10 ^b^  60 ^a,c,d,e,f,g,h,i^ | 4.8 |  |  |  |  |  |  |  |  |
|  |  |  |  | Annealing | 40 ^a,c,d,e,f,g,h,i^  50 ^b^ | 60 | 2.5 |  |  |  |  |  |  |  |  |
|  |  |  |  | Melting interval | 65 | 1 ^a,c,d,e,f,g,h,i^  15 ^b^ | 4.8 |  |  |  |  |  |  |  |  |
|  |  |  |  | Continuous | 95 | - | 0.02 | Acquisition 25 (/°C) | | |  |  |  |  |  |

^*^ Touchdown method, ^a^ *CDH1*, ^b^ *CDKN2A*, ^c^ *MLH1,* ^d^ *MGMT*, ^e^ MINT1*,* ^f^ MINT31*,* ^g^ *RUNX3,* ^h^ *miR-124a-3,* ^i^ *miR-34c*
